# Oral morphine versus transmucosal diamorphine for breakthrough pain in children: methods and outcomes: UK (DIPPER study) consensus

**DOI:** 10.1136/bmjspcare-2021-003278

**Published:** 2021-12-13

**Authors:** Emily Harrop, Christina Liossi, Liz Jamieson, Silke Gastine, Kate Oulton, Simon S Skene, Richard F Howard, Margaret Johnson, Katherine Boyce, Lorraine Mitchell, Satbir Jassal, Anna-Karenia Anderson, Richard D W Hain, Michelle Hills, Julie Bayliss, Archana Soman, Joanna Laddie, David Vickers, Charlotte Mellor, Tim Warlow, Ian CK Wong

**Affiliations:** 1 Helen and Douglas House Hospice, Oxford, UK; 2 Oxford University Hospitals NHS Foundation Trust, John Radcliffe Hospital, Oxford, UK; 3 School of Psychology, University of Southampton, Southampton, UK; 4 Department of Psychology, Great Ormond Street Hospital for Children NHS Foundation Trust, London, UK; 5 Research Dept of Practice & Policy, University College London School of Pharmacy, London, UK; 6 UCLH-UCL Centre for Medicines Optimisation Research and Education, London, UK; 7 UCL Great Ormond Street Institute of Child Health, London, UK; 8 Centre for Outcomes and Experience Research in Children's Health, Illness and Disability, Great Ormond Street Hospital for Children NHS Foundation Trust, London, UK; 9 Surrey Clinical Trials Unit, University of Surrey, Guildford, UK; 10 School of Biosciences and Medicine, University of Surrey, Guildford, UK; 11 Department of Anaesthesia and Pain Medicine, Great Ormond Street Hospital for Children NHS Foundation Trust, London, UK; 12 Department of Public Health and Primary Care, University of Cambridge, Cambridge, UK; 13 Flexicare Oxford & Abingdon, Oxford, UK; 14 Great Ormond Street Hospital Children's Charity, London, UK; 15 Rainbows Hospice for Children and Young People, Loughborough, UK; 16 Paediatrics, Royal Marsden Hospital, Sutton, UK; 17 Shooting Star Children's Hospice, Guildford, UK; 18 All-Wales Managed Clinical Network in Paediatric Palliative Medicine, Cardiff and Vale University Health Board, Cardiff, UK; 19 Martin House Hospice for Children and Young People, Boston Spa, UK; 20 Leeds Teaching Hospitals NHS Trust, Leeds, UK; 21 The Louis Dundas Centre, Oncology Outreach and Palliative Care, Great Ormond Street Hospital for Children NHS Foundation Trust, London, UK; 22 Sheffield Children's Hospital, Sheffield, UK; 23 Bluebell Wood Children's Hospice, North Anston, UK; 24 Evelina London Children's Hospital, London, UK; 25 Medical Director, Cambridgeshire Community Services NHS Trust, St Ives, UK; 26 East Anglia's Children's Hospices, Cambridgeshire, UK; 27 Bristol Royal Hospital for Children, Bristol, UK; 28 University Hospital Southampton NHS Foundation Trust, Southampton, UK; 29 Naomi House and Jacksplace, Winchester, UK; 30 Department of Pharmacology and Pharmacy, The University of Hong Kong, Pok Fu Lam, Hong Kong

**Keywords:** pain, paediatrics

## Abstract

**Objectives:**

No randomised controlled trials have been conducted for breakthrough pain in paediatric palliative care and there are currently no standardised outcome measures. The DIPPER study aims to establish the feasibility of conducting a prospective randomised controlled trial comparing oral and transmucosal administration of opioids for breakthrough pain. The aim of the current study was to achieve consensus on design aspects for a small-scale prospective study to inform a future randomised controlled trial of oral morphine, the current first-line treatment, versus transmucosal diamorphine.

**Methods:**

The nominal group technique was used to achieve consensus on best practice for mode of administration, dose regimen and a range of suitable pain intensity outcome measures for transmucosal diamorphine in children and young people with breakthrough pain. An expert panel of ten clinicians in paediatric palliative care and three parent representatives participated. Consensus was achieved when agreement was reached and no further comments from participants were forthcoming.

**Results:**

The panel favoured the buccal route of administration, with dosing according to the recommendations in the Association for Paediatric Palliative Medicine formulary (fifth Edition, 2020). The verbal Numerical Rating Scale was selected to measure pain in children 8 years old and older, the Faces Pain Scale-Revised for children between 4 and 8 years old, and Face, Legs, Activity, Cry and Consolability (FLACC)/FLACC-Revised as the observational tools.

**Conclusions:**

The nominal group technique allowed consensus to be reached for a small-scale, prospective, cohort study and provided information to inform the design of a randomised controlled trial.

**Key messages**

**What was already known?**

- No randomised controlled trials have been conducted for breakthrough pain in paediatric palliative care.
- Currently there are no standardised outcome measures for pain in paediatric palliative care.

**What are the new findings?**

- In the context of a potential prospective randomised controlled trial, the panel favoured the buccal route of administration, with dosing according to the recommendations in the Association Paediatric Palliative Medicine formulary (5th Edition, 2020).
- The verbal Numerical Rating Scale was selected to measure pain in children >=8 years old, the Faces Pain Scale-Revised for children 4-8 years old, and FLACC/ FLACC-R as observational tools.

**Key messages**

**What is their significance?**

- clinical
  - This will enable the provision of clinical data to support medicines for breakthrough pain.
- research
  - The findings will inform a future randomised controlled trial of oral morphine versus transmucosal diamorphine in children and young people with breakthrough pain.
  - The findings provide some preliminary evidence of how to address the research recommendation in The National Institute for Health and Care Excellence (NICE) Guideline NG61 (End of Life Care for Infants, Children & Young People) relating to the administration of medication for breakthrough pain in children.

## Introduction

Achieving rapid control of breakthrough pain, defined in this study as pain that occurs despite regular treatment with opioids and is severe enough to warrant additional opioids, raises significant challenges.^1–3^ It occurs in children and young people (CYP) receiving palliative care, is rapid in onset and usually lasts 20–30 min, yet the usual first line treatment is morphine by mouth that can take up to 30 min to work. In contrast, diamorphine given transmucosally (sublingually, intranasally or buccally) is an effective, rapidly absorbed, fast onset, needle-free analgesic that is easy to prepare and administer even in non-hospital settings.

**Figure 1:**
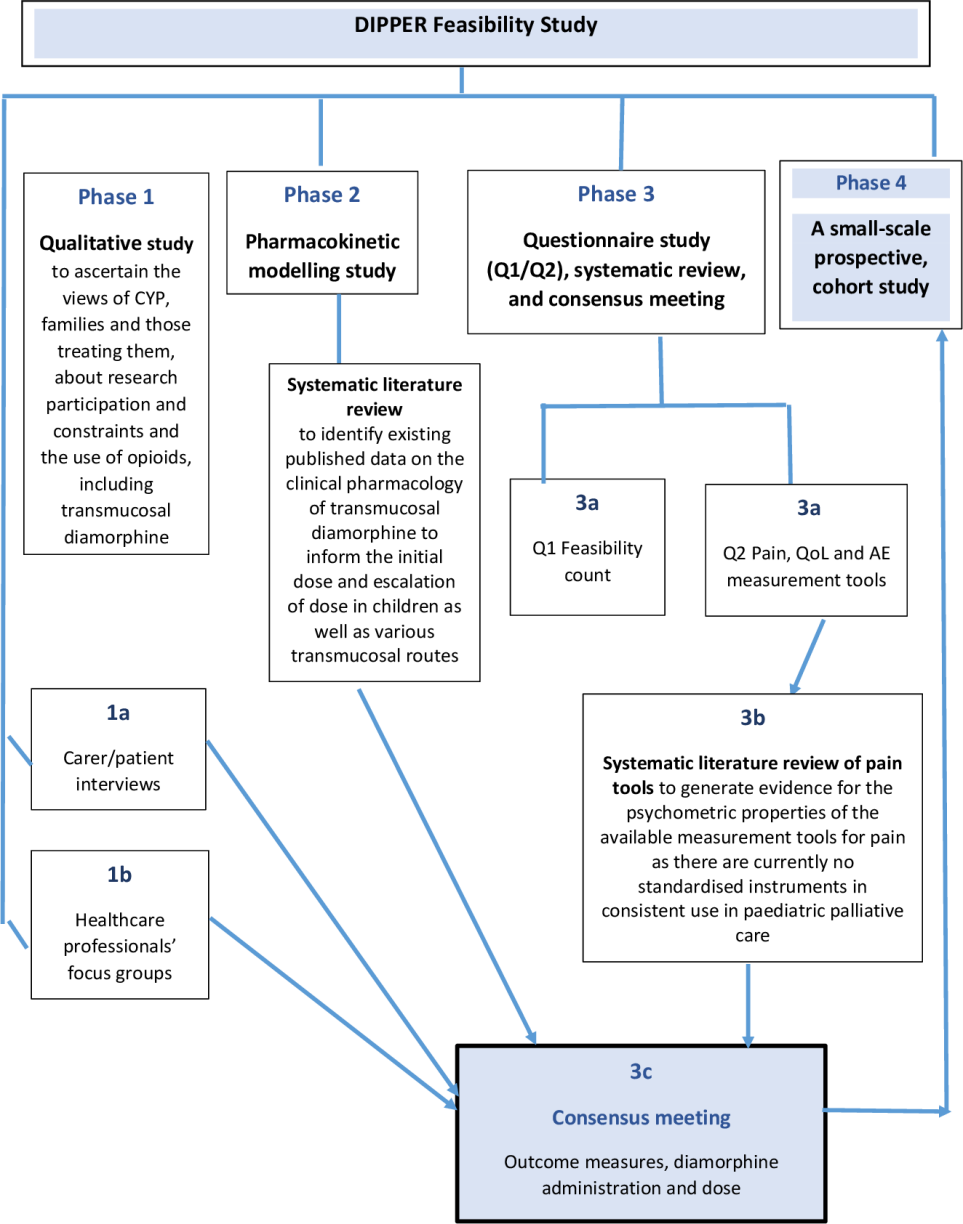
Phases of the DIPPER study(Feasibility of a randomised controlled trial of transmucosal diamorphine vs oral morphine for breakthrough pain in chidren and young people with life-limiting conditions). AE, adverse event; CYP, children and young people; QoL, quality of life.

The DIPPER study is a four-phase investigation of the feasibility of a randomised controlled trial (RCT) of transmucosal diamorphine (TDia) versus oral morphine (OM) for breakthrough pain in CYP with life-limiting conditions (figure 1). Data from phases 1 to 3 informed phase 4, a small-scale prospective study.

In this study, we report the findings of phase 3c, the aim of which was to achieve consensus from clinicians’ perspectives and experience on:

- Pain outcome measures in CYP receiving palliative care.
- Best practice of administration and dose regimen of TDia in CYP for breakthrough pain.

## Methods

### Participants

Ten principal investigators (PIs) representing paediatric palliative care (PPC) clinicians working across hospice, community and hospital care settings serving diverse cross-sections of the UK population, and three parent representatives attended the DIPPER meeting. Five members of the project team observed and facilitated the discussion.

### Setting

The meeting was held in London in November 2019 and participants were paid travel expenses. The day was split into two parts:

#### Presentation of preliminary findings from DIPPER

##### Phases 1a/1b families’ and clinicians’ experiences of OM and TDia, preferences regarding transmucosal route and perspectives in relation to taking part in a trial

LJ presented findings from three focus groups with healthcare professionals and from three preliminary parent interviews.^4^ OM was frequently used for breakthrough pain across settings whereas TDia was mainly used in hospices or given by community nurses, predominantly buccally. Healthcare professionals and families agreed that familiarity with the buccal route is due to experience with midazolam. Some focus group participants had experience of intranasal and sublingual administration. Perceived advantages of OM were ease and confidence in use and no requirement for additional training; disadvantages were slow onset, unpredictable response and potential unsuitability for some patients with gastrointestinal failure or other contraindications to enteral medications. Perceived advantages of TDia were quick onset and easy administration. Perceived barriers included lack of licensed preparations and prescribing guidance, and potential issues with availability, preparation, palatability, chances of it being swallowed, excess secretions or children closing their mouths tightly with buccal and sublingual routes. Factors that might affect recruitment to a trial were: patient suitability and perceived additional burden, CYP’s comfort at the time, trial design and logistics, staff time and clinician engagement.

##### Phase 2: pharmacokinetic modelling and systematic review of pharmacokinetic properties of TDia

SG presented literature findings on existing pharmacokinetic (PK) diamorphine data across formulations with very little data for TDia in CYP receiving palliative care. The review extracted information reporting metabolite data to draw conclusions of maturational effects on diamorphine PK and PK parameters for diamorphine and its metabolites, combining data from both adults and children, transmucosal and parenteral routes. A literature search on bioavailability and equianalgesic doses was also conducted; the Association for Paediatric Palliative Medicine (APPM) Master Formulary currently assumes an equianalgesic dose of 5 mg intravenous morphine to 3 mg intravenous diamorphine.^5^

**Table:** 

Morphine equivalence single dose	
**Analgesic**	**Dose**
Morphine subcutaneous/intravenous	5 mg
Diamorphine subcutaneous/intravenous	3 mg

[Bibr R5]

Ratios ranging from 1 to 4 are reported throughout the literature, with most publications reporting a ratio of around 2.

Bioavailability is dependent on formulation and site of administration. Kidd *et al* studied the bioavailability of nasal diamorphine compared with intravenous calculated through measurements of the active metabolite, morphine.^6^ Taking both bioavailability (47%) and an equianalgesic ratio of 2 into account, an approximate dose equivalence could be derived for TDia and intravenous morphine.

##### Phase 3b: systematic literature review of psychometric properties and feasibility of pain tools

Assessing pain in CYP is challenging due to diagnostic heterogeneity, varying types of pain and often a reduced ability to communicate verbally as a result of immaturity and developmental delay. CL presented the findings and recommendations of the systematic literature review. Thirty-four articles met the eligibility criteria and 22 pain assessment tools were examined. Evidence was limited and the methodological quality of included studies was low. No pain assessment tools have been validated in PPC settings. Balancing aspects of feasibility and measurement properties, the Faces Pain Scale-Revised (FPS-R) was recommended for self-assessment, and The Face, Legs, Activity, Cry and Consolability scale (FLACC)/FLACC Revised and Paediatric Pain Profile (PPP) were recommended observational tools in their respective validated age groups.^7–10^

#### Consensus discussions

We sought to achieve consensus on four areas:

1. Administration of TDia: buccal, intranasal or sublingual.
2. Dose regimen of TDia.
3. Outcome measures for pain.
4. Acceptability of taking part in the prospective study/RCT.

### Procedure

**Figure 2:**
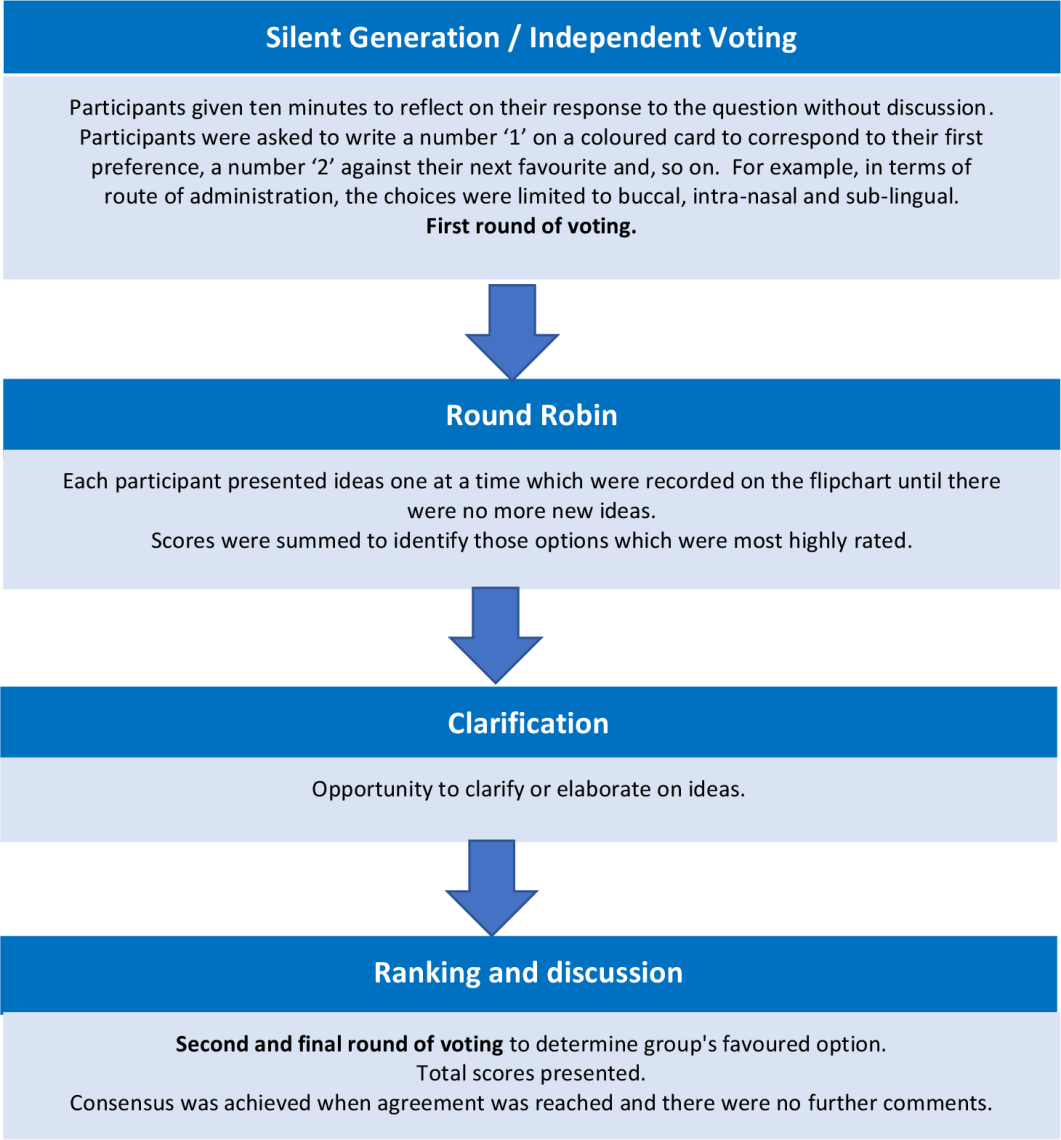
Nominal group technique procedure.

The sequence of events at the workshop was structured as shown in figure 2, we adapted the nominal group technique (NGT) to achieve consensus.^11 12^ This method has previously been used in health services research.^13–16^ All participants are given equal opportunity to contribute their own views before reflecting on those of others to reach consensus. Introductions and a brief explanation regarding the procedure of NGT was provided by the chair (CL), verbal consent was obtained to record the discussions, which were later transcribed.

### Analysis

Recordings were transcribed verbatim and analysed using thematic analysis to identify key experiences, perspectives, and points of agreement or disagreement within each discussion area. Furthermore, these data helped us to understand and contextualise the reasons for consensus and to inform recommendations for the prospective study and trial.

## Results

### Consensus discussion: 1: route of administration of TDia: buccal, intranasal, sublingual

Participants selected their first, second and third preference of mode of administration (table 1).

**Table 1:** Route of administration of TDis—round 1 voting

	First preference	Second	Third
Buccal	8	4	
Intranasal	2	5	
Sublingual		1	8

TDia, transmucosal diamorphine.

All participants agreed that sublingual was the least preferred route. The discussion then centred on barriers and facilitators to buccal and intranasal, such as potential side effects, ease of administration and acceptability (tables 2 and 3). Although there were advantages to both routes, the group felt staff and families would have more experience with the buccal route.

**Table 2:** Barriers and facilitators to the buccal route of administration

Barriers	Taste	Taste/palatability important, there could be a need to mask the flavour. Buccal midazolam does not taste very nice, and teenagers often refuse it until they get agitated; one clinician experienced this with young adults having buccal diamorphine. Young children do not mention taste, but maybe they are not asked.
	Mucositis/ulcerations	Concerns about mucositis or ulcerations, particularly with the cancer population having more modalities of treatment.
	Drooling	Buccal midazolam has a risk of increasing salivation.
	Consistency	Teenagers do not like the fizz of buccal/sublingual fentanyl, preferring the lolly stick, or the ones that dissolve.
	Flexible dosing	Flexible dosing is difficult. A device to administer prefilled, buccal diamorphine would be required to avoid making different strengths with different coloured bottles for different doses, as with buccal midazolam. Some clinicians used a licensed product for nasal use buccally.
	Administration	Some children purse their lips, making it impossible to administer. One PI had difficulties with administration in children on high dose antiepileptics who had gum hypertrophy. While a lot of mucous is exposed, buccal medicines slough off when administered and it is not possible to get it into the cheek itself.
Facilitators	Ease of use	Buccal was felt to be easier to use than intranasal as it is a squirt around an area, and is absorbed immediately after massaging it in. There is no need to coordinate with inhalation. However, as it is a liquid it would not be a problem in patients with a dry mouth. As with buccal midazolam, it is possible to spray on one side of the cheek, and if the dose is increased, the other cheek can be sprayed using the same dose and concentration.
	Experience of families and staff	Families have been taught how to give it and have experience of it. Many patients with multiple symptoms towards end of life are on buccal midazolam for agitation alongside pain relief. Some care teams use buccal diamorphine, and it would be easier than intranasal for them in terms of talking to families. Having one technique could mean fewer safety incidents.
	Information	There are already information leaflets, often pictorial, on delivery of buccal medication and instructions are on symptom management plans. Different instructions would be needed for delivery of an intranasal medicine.
	Applicability	Buccal was felt to be applicable to more children than intranasal. For very young children preprepared solutions cannot be used.

PI, principal investigator.

**Table 3:** Barriers and facilitators to the intranasal route of administration

Barriers	Risk of trauma	Many children have spasms and using an intranasal cannula in a moving nose would risk trauma more than buccal. Concern regarding trauma to the nasal cavity in patients with leukaemia, haematological or some metabolic patients with low platelet counts, where there may be increased nasal bleeding. Clinicians felt that oncologists would agree that that the intranasal route would be contra-indicated. In children with cancer who have low platelets, whether due to chemotherapy or because the tumour has advanced, and there is marrow replacement, a plastic cannula could not be used.
	Nasal congestion affecting absorption	Concerns around whether nasal polyps and nasal congestion may slow the absorption and how much would come out from squirting.
	Limited no of strengths	Ayendi (intranasal diamorphine) has only two strengths, sometimes requiring several sprays to be administered.
	Cumulative sensitivity/nasal irritation	Concern recumulative sensitivity to the nose. However, no local effects had been experienced by a patient with Epidermolysis Bullosa who had used it for nearly 2 years. Also, intranasal fentanyl is used in the USA; no nasal irritation has been found. In the trials with Ayendi in trauma there was no aversive response when patients were given a repeater dose, but they did not enjoy it.
	Practical considerations	Respiratory support may have to be removed to deliver pain relief intranasally in some children.
	Acceptability	Some young children do not like the nasal influenza vaccine. Parent representatives felt that children might be fearful seeing something coming towards their face.
Facilitators	Quicker access to brain?	It was felt that the intranasal route potentially had quicker access to the brain, but there was uncertainty whether there was evidence for this.
	Speed of administration	Parent representatives said they would instinctively opt for the nasal route if their child was in pain as they thought it would be quicker than via the mouth.
	Useful for patients with oral aversion	Useful for patients with oral aversion and for those with epidermolysis bullosa who may only require it once a day for dressing changes.
	Multiple dose applicator	Ayendi (intranasal diamorphine) has a multiple dose nasal applicator, rather than a single dose syringe, which assists dispensing for families.

Having heard parent representatives’ views of the intranasal route potentially being quicker, one PI changed their first preference from buccal to intranasal in the second round of voting (table 4). Two respondents put a ‘1’ down for both buccal and intranasal, which was not permitted, but they explained that it would depend on the circumstances.

**Table 4:** Route of administration of TDia-round 2 voting

	First preference	Second	Third
Buccal	11	11	
Intranasal	1	1	

TDia, transmucosal diamorphine.

**Table 6:** Barriers and facilitators to Self-Report Pain Scales

Barriers	General	Population	One participant only voted for behavioural observation scales as 85% of children in their service are cognitively unable to engage.
		Time in trial	Clinicians expressed different concerns: some felt that if children were in the trial for a short time, they would need to be familiar with a scale. Some felt that if children were using something different for a long time that might be confusing whereas others felt it could be hard to change for a short time, but it could be incorporated for a few months. However, one advantage of using a different scale is that it would minimise contamination
		Importance of good psychometric properties	Clinicians queried whether it was necessary to have a tool with good psychometric properties, particularly if children will not engage with it. They felt that it is important to be pragmatic because children do not want to talk when they are having a breakthrough pain episode.
		Education	Community nurses may use different scales, so may require education.
	Specific scales	vNRS	Difficulty with the concreteness of the question: “ ….*where is your pain*”? on the scale as some respond: *“(t’s) in my back*”, particularly young people with a cognitive impairment
			Concern over whether older children would feel they were being treated as babies using faces (the faces do not look like babies).
Facilitators	Specific scales	FPS-R	For children who can self-report their pain, it has to be quick, so numbers work better and because it is administered verbally does not require any materials other than a piece of paper to note the response.
		FPS-R	For the younger children, it was felt that it would be easy to change to the FPS-R as they use the Wong and Baker faces^19^ in oncology from diagnosis and it is on the ward at some sites so would help with staff engagement.

FPS-R, Faces Pain Scale-Revised; vNRS, verbal Numerical Rating Scale.

Clinicians felt that different scenarios might require different routes, but all agreed that the buccal route would make the study more acceptable. Clinicians agreed they would support the buccal route for this prospective study on the grounds that there is more experience of it, but that there might be reasons why the intranasal route would be more suitable for an individual patient. At the time of this study there was an intranasal product on the market.

Consensus was reached that the buccal method would be used for the prospective study.

### Consensus discussion 2: guidance on the dose regimen of TDia in CYP for breakthrough pain

Clinicians were asked for their opinions on dosing; this was a variation to the NGT as there was no voting on selected choices. It was felt that the proposed study should not deviate too much from current guidelines for buccal and intranasal doses of diamorphine as per the APPM formulary.^5^

Currently, the guidelines recommend the same doses for both intranasal and buccal routes. Historically some clinicians have assumed that absorption of TDia was approximately 100% as there is no evidence to confirm or deny this and so the doses used have reflected that. Similarly clinicians felt that ‘modelling of one-to-one’ (1 mg intravenous morphine=1 mg TDia) would be a safe starting dose when considering potentially important side effects, and it was felt important to ‘play safe’ when scaling adult doses. However, there should be an option to escalate doses if the response was inadequate. All agreed on the safer starting dose; to be titrated up to clinical effect. However, more evidence was needed about transmucosal absorption and modelling would be required. In terms of frequency, all felt it would be helpful if the raw data could be obtained and modelled for peak absorption. This was also an argument in favour of using buccal, as it would provide a comparator as there is no empirical evidence to prove that buccal is equivalent to intranasal.

Consensus was reached that the APPM Master formulary recommendations for dosing would be used with the dose adjusted according to clinical response.^5^

### Consensus discussion 3: outcome measures for clinical trials in CYP for pain

Participants were asked to select their first, second and possibly third, preference of pain scale for (1) self-report (for patients who are able to) and (2) behavioural observation (for non-verbal children or who have cognitive impairments) for measuring breakthrough pain.

#### Self-report scale

One option for a self-report scale was the FPS-R that had been presented earlier,^7^ or participants could vote for their own preference, or state ‘no preference’.

Voting reflected the importance of age/developmental stage in choosing an appropriate scale (table 5). Children as young as four can use the FPS-R up to eighteen years.^7^ A verbal Numerical Rating Scale (vNRS) could be used for children aged 8 and older.^17^ The Visual Analogue Scale (VAS) has the best evidence for acute pain in stated age group, but no evidence in palliative care.^18 19^ All pain scales assess acute (mostly postoperative) pain, but there is research using the VAS and NRS for chronic pain.

**Table 5:** Self-Report Scale-voting

	First	Second	Third
Numerical Rating Scale	4 (one person said >10 years)		
Faces Pain Scale Revised	4		
Various Faces Rating Scales	3 (2 said 4–10 years old)		
Clinician observations	1		
No preference	1		

Clinicians reported site variability in the use of scales, some who were administering opioids were not routinely using pain assessment tools. Some used them to determine the appropriateness of drugs and treatment response; others obtained qualitative reports from patients or parents. Personalised behaviour scales were used for complex, non-verbal children or young people with good/bad day behaviours.

Specific points raised in favour of or against self-report scales are shown in table 6.

The group were asked to decide whether they preferred one unified scale or different scales for different age groups, which would require different instructions. One option could be to have the FPS for everybody up to 18 years, or to have the FPS for younger children and the NRS for older children. After discussion, it was agreed that for a trial, scoring would need to be standardised and families would accept that.

Consensus was reached to use the vNRS for those aged 8 and older and the FPS-R, for children aged 4–7 years. For those with learning disabilities who are verbally communicative, but who might have difficulties with scoring, clinicians could judge whether to use the FPS-R, irrespective of age.

#### Behavioural observation measure

Participants remarked that behavioural observation measures are not pain specific, so staff observe and rate distress, but may not know if the child is in pain. Options included the FLACC and PPP. Initial voting found consensus to use the FLACC as the behavioural measure^8–10^ (table 7).

**Table 7:** Voting—behavioural observation measure

	First	Second	Third
FLACC	8		
Paediatric pain profile	1		
Behavioural	1		
No preference	1		
None	1		

FLACC, Faces, Legs, Activity, Cry and Consolability.

Some clinicians mentioned that many children may not be able to demonstrate the behaviours in the FLACC, for example, move their legs or cry out, and personalised FLACCs, written with parents’ input, are used. However, adapting the FLACC for research purposes (as opposed to clinical needs) would not be possible and anything that limits scoring would have to be reported. FLACC would also be of limited use in a patient who was not always conscious, perhaps close to end of life. A specifically modified FLACC was studied in paediatric intensive care where it was compared with the Comfort-B scale.^20^ The latter scale was a more reliable measure of children’s sedation than bedside subjective assessment and gave more information about sedation than the modified FLACC scale. However, concurrent validity for assessment of pain was supported for both scales. The modified FLACC showed construct validity for measuring pain.

### Consensus discussion 4: issues raised regarding the prospective study/ trial

This phase involved a discussion of issues to reach a decision, without voting.

#### Quality of life and adverse events as outcome measures

It was agreed that it would be difficult to measure quality of life reliably as multidimensional QoL measures look at QoL over weeks. However, in the main trial patients would be followed up for longer and a health-related quality-of-life (HRQOL) measure would be included. A systematic review by Coombes *et al* on HRQOL outcome measures in PPC found that there is no ‘ideal’ outcome assessment measure, and an existing measure could either be adapted or more individualised patient-centred outcome and experience measures developed.^21^ The PedsQL Generic Core Scale is widely validated and contains various disease-specific modules that can be administered alongside the core scale.^22^ Some clinicians used the PaedsQL, but it is not specific for palliative care. Clinicians felt that it could also be quality of life of the family, or a global impression using a visual scale or verbal reporting although, again, this would not be meaningful after a few episodes of breakthrough pain. It was also feared that more questionnaires used might mean less compliance.

Standardised checklists are not being used for recording adverse events in clinical practice; adverse events are recorded in clinical notes or internal reporting systems. However, these will have to be reported in clinical trials in a standardised way.

#### Numbers of patients

Each centre would recruit two patients, ideally one requiring buccal diamorphine and one requiring OM for breakthrough pain, to take part in the observational, prospective study. As the prospective study is not a drug trial, and is purely observational, the treatment decision will be based on patient need and normal practice.

#### Number of pain episodes to measure and timing

The peak effect of buccal diamorphine is about 10 min, and Oramorph 20–30 min. After discussion, it was agreed to measure the first four episodes of breakthrough pain and, for each episode, pain to be assessed at four time points: T0: baseline prior to pain relief, T10: 10 min, T30: half an hour, T60: an hour, that is, 16 measurements per patient. Data collection will end after recording scores for four episodes or after 1 month, irrespective of the number of episodes.

#### Trial design

Clinicians considered two options: patients acting as self-controls (crossover in the same patient). As there is more than one episode, this is statistically more powerful, the required sample size is smaller and a shorter time is needed.^23^ Patients receive dummy treatment and an active treatment (buccal diamorphine or OM) for each episode, but do not know which is active. However, participants would need to be able to swallow to take OM, and to take something buccally simultaneously would be an additional burden. The second choice is to accept simple randomisation (OM or buccal DM). Clinicians felt that the first option would be empowering as it would invite families to say which is the better of the two treatments, even though it might be more complex to explain. However, they queried whether patients with enteral feeding tubes could take part. Also, as OM is in the body for 2–3 hours a 4-hour wash-out would be needed, and they would need guidance in the trial about what to do if children have pain. Clinicians also felt that the trial should inform across the age range, including <2 years.

#### Practical issues in administration and scoring

Some clinicians were concerned about trialling a new method of administration at home and felt it would be preferable to keep patients in the hospital/hospice, at least for the first two episodes.

It was felt that families who take part will complete the pain scoring, but it should be minimal, with simple instructions. Other options included; a researcher or clinicians being trained and paid to recruit and train families, or preferably a research nurse through the local clinical research network. It would be possible for some patients/ families to do it at home as it is self-controlled, and the bias is the same. Other suggestions included an app that beeped as a reminder or the family ringing a nurse to document the score.

One issue was raised about patients on patient-controlled analgesia for acute pain being able to take part when they are being weaned onto Oramorph as required. Another issue was that patients may not always be treated with opioids, particularly non-oncology patients. Children with breakthrough pain management issues are often prescribed opioids, many of whom are non-communicative.

#### Acceptability questionnaire for the prospective study

At the end of data collection, patients/carers will be given a questionnaire about the acceptability and experience of using OM or buccal diamorphine, and the time needed to collect the data. Clinicians felt that the questionnaire should be very short and address how tolerable it was to take OM or buccal diamorphine with an opportunity for free text reporting of their experience including burden and feasibility. Some felt that the questionnaire should be given when the study is fresh in people’s minds; others opted for 24–48 hours afterwards, to allow reflection but the child may have had many pain episodes or may have deteriorated in that time. Another suggestion included having stickers or thumbs up/down for children. Parent representatives felt that participants should be thanked and informed as to how and where they can access the results.

## Discussion

### Principal findings

The National Institute for Health and Care Excellence (NICE) Guideline NG61 (End of Life Care for Infants, Children & Young People) identified a paucity of research evidence relating to the administration of medication for breakthrough pain in children, leading to a specific research recommendation.^24^ The NGT method enabled agreement on route of administration, dosing and standardised outcome measures for breakthrough pain to be reached for a small-scale prospective cohort study as well as information to inform the design of a future RCT (table 8). There was a high degree of congruity among the participants.

**Table 8:** Consensus decisions

Route of administration	Buccal
Dosing	According to APPM guidelines Edition 5 (2020)
Pain tools	Self-report: vNRS for those aged 8 and older and FPS-R for children aged 4–7 years. Observational: FLACC for 2–3 years; cognitively unable (and/or FLACC_R with additional behavioural descriptors)

APPM, Association for Paediatric Palliative Medicine; FLACC, Faces, Legs, Activity, Cry and Consolability; FPS-R, Faces Pain Scale-Revised; vNRS, verbal Numerical Rating Scale.

While many of the contributions made by the PIs at the meeting reflected their personal practice or their own opinion, the PIs who attended were a fair representation of senior clinicians currently working in the small field of PPC in the UK. There was familiarity with the buccal route, which could increase acceptability, similar to findings in the focus groups with healthcare professionals with experience of palliative care for CYP in both primary and secondary care.^4^ They felt that there was limited experience of using intranasal diamorphine (mainly in Accident & Emergency for trauma/fractures) and sublingual diamorphine. Experience with buccal midazolam, approved by NICE (2012) as a first line drug in children and young adults who develop prolonged convulsive seizures, would increase professionals’ confidence using this route, and increase acceptability, as well as ease of administration for the family and a lack of need for training.^25^

Clinicians opted to use the APPM Master Formulary (based on published research and consensus expert opinion) recommendations for dosing rather than having a standardised dose regimen.^5^

Pain assessment tools are vital to inform clinicians in relation to pain management decisions. There are no pain assessment tools validated specifically for CYP in PPC settings. A range of validated tools is required to meet the different developmental and communication needs of the PPC population that are easy-to-use. Our systematic review found few studies that focused on the validation of these tools in CYP with PPC needs. It found several scales that demonstrated high levels of feasibility, but they were not recommended due to the lack of validation evidence in CYP with LLCs or PPC settings.

An overview of systematic reviews of pharmacological interventions for chronic pain in children found there were no RCTs for pharmacological interventions in children with cancer-related pain.^26^ However, both the focus groups and consensus meeting have highlighted some important issues to address in terms of designing a trial of OM vs TDia, including the burden to families, timing in terms of the patient’s trajectory and research support to centres.

### Strengths and limitations

We believe this is the first study to run a consensus meeting with senior clinicians in PPC to ascertain their experience of TDia, best ways to measure pain relief and their thoughts about a future RCT. A disadvantage of NGT is that the method is very structured and only deals with one issue at a time.

### Implications for practice and future research

Further research is needed to validate current pain measures in PPC and there is also a need to develop a breakthrough pain specific measure.^27^ Regarding general measures, it would be beneficial to develop a core outcome set for PPC similar to adult initiatives to optimise clinical decision making and allow for accurate comparison between studies in systematic reviews and/or in meta-analyses.^28^

## Data Availability

All data relevant to the study are included in the article.
